# Integrin α6β4 in Colorectal Cancer: Expression, Regulation, Functional Alterations and Use as a Biomarker

**DOI:** 10.3390/cancers12010041

**Published:** 2019-12-21

**Authors:** Jean-François Beaulieu

**Affiliations:** 1 Laboratory of Intestinal Physiopathology, Faculty of Medicine and Health Sciences, Université de Sherbrooke, Sherbrooke, QC J1H 5N4, Canada; Jean-Francois.Beaulieu@USherbrooke.ca; Tel.: +1-819-821-8000 (ext. 75269); 2 Centre de Recherche du Centre Hospitalier Universitaire de Sherbrooke, Sherbrooke, QC J1H 5N4, Canada

**Keywords:** colorectal cancer, integrin α6β4, ITGA6, ITGB4, alternative splicing, expression, function, biomarker

## Abstract

Integrin α6β4 is one of the main laminin receptors and is primarily expressed by epithelial cells as an active component of hemidesmosomes. In this article, after a brief summary about integrins in the gut epithelium in general, I review the knowledge and clinical potential of this receptor in human colorectal cancer (CRC) cells. Most CRC cells overexpress both α6 and β4 subunits, in situ in primary tumours as well as in established CRC cell lines. The mechanisms that lead to overexpression have not yet been elucidated but clearly involve specific transcription factors such as MYC. From a functional point of view, one key element affecting CRC cell behaviour is the relocalization of α6β4 to the actin cytoskeleton, favouring a more migratory and anoikis-resistant phenotype. Another major element is its expression under various molecular forms that have the distinct ability to interact with ligands (α6β4 ± ctd) or to promote pro- or anti-proliferative properties (α6Aβ4 vs. α6Bβ4). The integrin α6β4 is thus involved in most steps susceptible to participation with CRC progression. The potential clinical significance of this integrin has begun to be investigated and recent studies have shown that *ITGA6* and *ITGB4* can be useful biomarkers for CRC early detection in a non-invasive assay and as a prognostic factor, respectively.

## 1. Introduction

**Figure 1:**
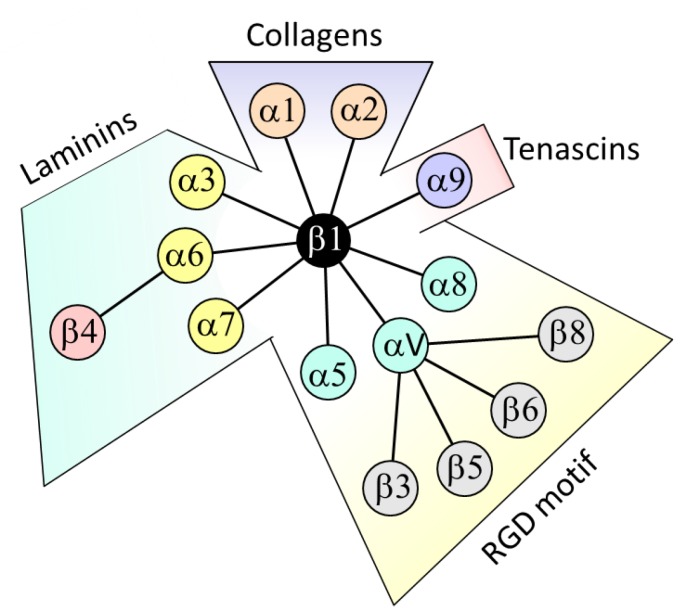
Integrins expressed in intestinal epithelial cells under normal or pathologic conditions. Integrins are classified according to their preferential ligands: collagens for α1/α2 coupled to β1, laminins for α3/α6/α7 coupled to β1 or β4, tenascin for α9β1 and RGD-containing ligands (fibronectin, osteopontin and vitronectin) for α5/α8 coupled to β1 and αV coupled to β1/β3/β5/β6/β8.

Epithelial cells lie and attach to the basement membrane, a specialized network of extracellular matrix molecules that comprise specific collagens, proteoglycans and exclusive glycoproteins such as laminins as well as non-exclusive ones such as fibronectins. Cell adhesion is mediated by membrane receptors specific for these glycoproteins. In the intestine, there are a number of these receptors that have been identified for epithelial cells including dystroglycan and the 37/67 kDa laminin receptor [1] as well as many that belong to the integrin family [2,3]. Integrins are transmembrane αβ heterodimers that function as bidirectional signal transduction mediators acting as mechanosensors and participating in the regulation of main cell functions such as adhesion, migration, proliferation, survival and differentiation [4,5]. The gut epithelium expresses a wide variety of integrins as well as their corresponding ligands. Figure 1 illustrates the various heterodimers identified and their extracellular ligands.

It is noteworthy that while some of these integrins are mostly associated with the differentiated state in the intestinal epithelium cells such as α3β1 and α7β1, many are predominantly expressed by the proliferative cells at the base of the epithelial cells of the crypts in both the small and large intestines [2,3,6] and, more importantly in the context of this review, in primary and/or metastatic colorectal cancer (CRC) cells. This has been well documented recently for the α1β1 integrin, a collagen receptor. While exclusively expressed in the proliferative compartment in the normal colon, α1β1 was found to be overexpressed in CRC primary tumour and cell lines [7,8] and knockdown of α1 subunit expression resulted in the reduction of cell proliferation and tumour growth [9]. Integrin α9β1 is another interesting case. Expressed in the proliferative cells in the normal gut at the fetal stage but not in the adult, its resurgence in CRC cells both in primary tumours and in vitro is indicative of an oncofetal pattern of expression [10,11]. Two of the RGD-dependent integrins, α5β1 and αVβ6, appear to contribute to the metastatic process of CRCs [12,13]. Interestingly, the β6 subunit is expressed at very low levels in epithelia under normal conditions but is detected in both CRC primary tumours and metastasis where it acts through various mechanisms and systems to promote cancer progression [12]. However, there are exceptions. For instance, α8β1 which has also been shown to promote intestinal cell migration and proliferation in normal cells [14] appears to be no longer expressed by CRC cells [15]. Further investigation using normal intestinal cells revealed that this integrin acts as an anoikis-sensitizing trigger factor while the repression of its expression abolished this checkpoint [15]. Conversely, its reintroduction into CRC cells restores sensitivity to anoikis [15]. The αVβ3 integrin was also reported to act as an anoikis-sensitizing integrin for CRC cells [16,17]. These examples illustrate the crucial importance of the integrins as extracellular matrix molecule receptors involved in CRC cell progression, some susceptible to directly act in its promotion such as α1β1, α5β1 and αVβ6 while others act as preventive checkpoints such as α8β1 and αVβ3 or favour quiescence and differentiation rather than proliferation such as α3β1 and α7β1. These effects are also context-dependent over the entire process, some acting at early stages of cancer development while, as mentioned above, others are more likely to act at a later stage such as in the metastatic process. Some integrins likely exert dual promoting/suppressive influence on CRC development and progression [18]. In this context, integrin α6β4 is one of the most intriguing cases since its different role in CRC may in part derive from it ability to assemble hemidesmosomes, a very stable cell-extracellular matrix adhesion structure, and its potential to modulate oncogenic cell signalling events when released from hemidesmosomes as well as its α6A/α6B variant composition. While it belongs to the laminin-binding integrin subset, which also includes α3β1, α6β1 and α7β1 [2,19,20], α6β4 is characterized by a large β subunit and its predominant expression is in parenchymal cells such as the epithelial cells that line the luminal surface of the colonic crypts. 

In this review, I will first present studies reporting increased expression of the β4 integrin subunit in CRC lesions and potential mechanisms accounting for the regulation of its expression. Through its unusual long cytoplasmic domain, the β4 subunit is also subject to relatively complex inside out and outside in signalling regulation that can affect both cellular adhesion and migration. β4 can only dimerize with the α6 subunit. In epithelial cells, this association appears exclusive since most α6, if not all, is associated with β4. The α6 subunit is subject to alternative splicing, generating two potential variants called α6A and α6B. In contrast to other tissues that predominantly express one or the other, the two variants are expressed in the epithelium of the small and large intestines but in distinct compartments, α6B being restricted to the quiescent differentiated cells while α6A is predominant in the normal proliferative cells required for the renewal of the digestive epithelium as well as in cancer cells. Progress concerning the mechanisms leading to α6A (*ITGA6A*) expression in neoplastic cells and its role in CRC progression will also be presented. Finally, as expected from these data, *ITGA6A* and *ITGB4* expressions in CRC primary tumours have been explored as prognostic factors; l will also present rationale for using *ITGA6* variants as biomarkers and data that confirm their validity for identifying patients with CRC lesions in a non-invasive mRNA-based stool test. 

Please note that as per convention, we are using the α6/β4 denomination for the proteins and *ITGA6/ITGB4* for the genes/transcripts. 

## 2. β4 Integrin Subunit (*ITGB4*) in CRC

### 2.1. Expression

The expression of the integrin β4 subunit in CRC has been the subject of a number of studies. While most of these have shown that β4 is uniformly distributed at the basal side of the colonic epithelium, its level of expression in primary CRC tumours was initially found to be quite variable depending on the study and antibody used, being found to be reduced or lost [21], maintained [22] or increased [23,24]. Increased expression was finally confirmed using a panel of distinct anti-β4 antibodies and validated at the transcript level [25]. 

The use of this panel of anti-β4 antibodies including two directed against extracellular epitopes and two targeting the cytoplasmic COOH-domain revealed interesting features. Indeed, in the normal small intestine and colon, cells of the crypts expressed a co-translationally processed form of β4 lacking the cytoplasmic terminal domain (β4ctd-) [26] so that only antibodies directed to the extracellular domain stained epithelial cells of the colonic crypts [25]. In contrast, the differentiated cells of the small intestinal villi and colonic surface epithelium expressed normal β4ctd+ suggesting that the β4ctd- form is related to the proliferative and less differentiated cells of the renewing intestinal epithelium [25,26]. Unexpectedly, colonic cells from both primary CRC and established adenocarcinoma cell lines expressed the β4ctd+ form, suggesting that this processing of β4 is lost or at least downregulated in neoplastic cells [25]. The potential impact of this is addressed below. 

### 2.2. Regulation of Expression

Integrin β4 subunit expression appears to be mainly regulated at the transcript level but the mechanism of its overexpression in CRC cells is still incompletely understood. There have been a number of studies that have reported specific potential interactions of transcription factors with the *ITGB4* promoter since its cloning [27] in a variety of cell types including RUNX1 [28], JUN [29] and KLF4 [30]. In CRC cells, MYC was the first transcription factor identified that promotes *ITGB4* transcription [25] but recent studies have found others such as ZKSCAN3 [31] and FOSL1 [32] that need to be considered. Epigenetic regulation of *ITGB4* expression has also been suggested after identification of microRNAs that may target the *ITGB4* transcript such as miR-21 [33] and miR-335-5p [32] as well as hypomethylation of the *ITGB4* promoter [32]. 

### 2.3. Change in Functionality

In normal epithelial cells, the β4 subunit is one of the key components of hemidesmosomes, a specialized adhesive structure found in epithelia. Most integrin β subunits bear short cytoplasmic domains smaller than 50 amino acids able to interact with actin filaments through cytoplasmic linker proteins such as talin and vinculin that are also used as scaffolding for the recruitment and activation of intracellular signalling pathways [34]. In contrast, β4 has a 1000 amino acid cytoplasmic domain composed of distinct sequences not found in other β integrins: a Calxβ motif adjacent to the plasma membrane, two pairs of fibronectin type III domains, a connecting segment and a COOH-terminal end domain [35]. In hemidesmosomes, α6β4 mediates the intracellular interaction with cytoskeletal keratins through various plakins such as plectin and the extracellular interaction with laminins, preferentially laminin-332 [35]. However, hemidesmosomes are dynamic structures that need to be dismantled to allow cell migration and other cell dynamic functions. There are a number of mechanisms that have been suggested for the release of α6β4 from the hemidesmosomes [18] mainly the phosphorylation of the β4 cytoplasmic tail in response to receptor tyrosine kinase activation by growth factors [18,36]. Upon release, α6β4 switches its association with cytokeratin to relocalize with actin filaments favouring the formation of motility structures [36]. In this context, it is worth mentioning that the processing of β4 into β4ctd- in normal proliferating intestinal cells impairs the ability of α6β4 to bind to laminin [26], which by increasing susceptibility to anoikis may represent an additional checkpoint mechanism for preventing aberrations in the permanent cell population responsible for epithelial renewal in the intestine [37]. 

**Figure 2:**
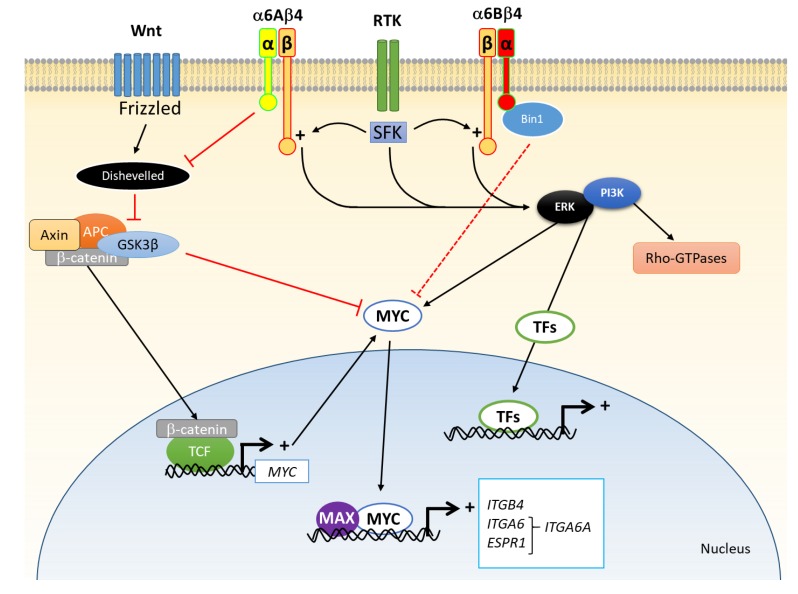
Some of the signaling pathways regulated by integrin α6β4 in colorectal cancer cells. First is the cooperation between receptor tyrosine kinases (RTK), which via Src family of kinases (SFK) trigger phosphorylation of the cytoplasmic domain of β4 to activate the ERK and PI3K signaling pathways, which in turn regulate various cellular functions such as migration, proliferation and survival by the modulation of specific transcription factors (TFs) and activation of the Rho-GTPases. Second are the regulatory effects of the α6 subunit on cell proliferation where α6A promotes the Wnt/β-catenin pathway and the expression of downstream effectors such as MYC while α6B appears to inhibit MYC activity by a possible interaction with the protein bridging integrator 1 (Bin1). MYC appears also as one key regulator of integrin expression since both *ITGB4* and *ITGA6* have MYC-responsive elements in their promoters as for *ESPR1* that encodes a splicing factor that regulate *ITGA6A* expression. Adapted from [18,36,39,40].

In carcinoma cells, numerous studies have demonstrated that α6β4 promotes cell motility and invasive behaviour rather than stable anchoring to the extracellular matrix via a mechanism related to the one used for cell migration in wound healing in normal cells [36]. Since the discovery that α6β4 promotes colonic carcinoma cell invasiveness by activating the PI3K pathway [38], there have been a number of studies that have contributed to better documenting the potential of integrin α6β4 to regulate multiple signal transduction cascades involved in the promotion of cell proliferation, migration, invasion and suppression of anoikis [18,36,39]. While I invite the reader to refer to these seminal reviews [18,36,39] for further details about the multiple signalling transduction cascades that can be activated by α6β4 upon binding to its ligand, a few crucial elements are worth mentioning herein in the context of colorectal cancer (Figure 2). First, is the ability of α6β4 to synergistically cooperate with oncogenic receptor tyrosine kinases such as those of the epidermal growth factor receptor family and c-Met, which via their downstream effectors, the Src family of kinases, trigger the phosphorylation of tyrosine residues in the cytoplasmic domain of the β4 subunit to enhance the signal [34]. Second are the mechanisms by which α6β4 promote migration and invasion, which include the activation of the small GTPase RhoA and the up-regulation of the metastasis-associated protein S-100A4 [36]. Third is the apparent dual influence of α6β4 on cell survival. In most cell types, cell survival requires an α6β4-dependent PI3K activation and blocking α6β4-mediated adhesion trigger apoptosis [39]. However, using the RKO colon carcinoma cell line, Mercurio’s team showed that the ability of α6β4 to promote or inhibit apoptosis depends on the p53 cell status. Furthermore, the loss of the β4ctd- form in carcinoma cells, which strengthens α6β4-laminin interactions, and thus outside-in signalling, may also account for these effects.

## 3. α6 Integrin Subunit (*ITGA6*) in CRC

### 3.1. Expression

As mentioned above, the integrin α6 subunit is the only possible dimerization partner for β4. *ITGA6*, as for *ITGA3* and *ITGA7* thought to have been derived from a common ancestor [20], is subject to alternative splicing of its exon 25, leading to the generation of two variants of the α6 subunits, α6A and α6B, that are distinct in their unique cytoplasmic sequences [20]. Although the α6 variants were reported and characterized more than 25 years ago and are likely to participate in distinct signalling cascades [41], the relationship between variant expression and distinctive cell functions has remained elusive especially in tumorigenesis [18]. 

As for many other epithelial cell types, intestinal cells can express both variants [41]. Closer analysis of α6A and α6B distribution in the intact normal human gut with a panel of anti-α6 antibodies revealed that α6A was predominantly detected in the proliferative cells of the small and large intestine while the α6B subunit was more restricted to the quiescent/differentiated cells lying on the villus and surface epithelium of the two intestinal segments [42,43]. Predominant expression of α6A in proliferative/undifferentiated cells and its gradual replacement by the α6B subunit in differentiating cells has also been confirmed in intestinal cell models [43]. 

Expression of the α6 variants was investigated in CRC in situ and in adenocarcinoma cell lines. In CRC primary tumours, although most carcinoma cells expressing both α6A and α6B a clear loss of α6A/α6B segregation was observed [43]. Quantitatively, a clear increase in total *ITGA6* transcripts was noted in CRC as compared to the corresponding resection margins and this increase was attributed to an up-regulation in *ITGA6A* expression, with total levels of *ITGA6B* remaining comparable to those of the resection margins [44]. Consistently, all colorectal adenocarcinoma cell lines tested under proliferative conditions showed predominant *ITGA6A* expression. 

### 3.2. Regulation of Expression

Information about transcriptional regulation of *ITGA6* expression is still limited. Among the consensus binding sites for the transcription factors SP1, NF-kB, AP1 and MYC identified on the *ITGA6* promoter [45], only SP1/SP3 have been confirmed [46]. Considering the fact that MYC expression is up-regulated in a large proportion of CRC [47,48] and that data indicating that MYC controls the expression of many genes in CRC cells including several key integrin subunits [40], which comprise *ITGB4* [25], MYC represents a potential regulator of *ITGA6* transcription. Pharmacological inhibition of MYC activity as well as molecular manipulations of intracellular MYC levels has shown a direct concordance between MYC levels and *ITGA6* expression in intestinal cells while chromatin immunoprecipitation assays have confirmed the functionality of the MYC binding site on the *ITGA6* promoter in the intestinal context [49]. 

Since neo *ITGA6* is expressed under the form of *ITGA6A* in CRC cells [24], the mechanism responsible for the up-regulation of this spliced form was further investigated. Alternative splicing of *ITGA6* has been relatively well studied and at least 10 splicing factors have been identified under various contexts but not in CRC cells. Among the various factors tested, the epithelial splicing regulatory protein 2 (ESPR2) was identified to be the main splicing factor responsible for *ITGA6A* expression in CRC cells [49]. It is noteworthy that ESPR1 was found to be responsible for *ITGA6* splicing in breast cancer cells [50] but in contrast to *ESPR2* that was found to be up-regulated by MYC, *ESPR1* was not modulated in CRC cells by this factor [49]. Further identification of MYC as a direct activator of *ESPR2* active transcription indicates that MYC regulates both *ITGA6* and *ESPR2* expression resulting in the specific up regulation of *ITGA6A* in CRC cells [30]. 

### 3.3. Change in Functionality

It is interesting to note that the cells of the crypt proliferative compartment, which predominantly express α6A/*ITGA6A* [42,43], are also the cells that express MYC in the normal colon [9]. Experimental studies using a normal intestinal epithelial crypt cell line showed that MYC regulates *ITGA6A* expression [49]. These data suggest that the MYC-dependent regulation of *ITGA6A* is a phenomenon occurring in the normal intestine, which appears to be exploited throughout the neoplastic process. 

Indeed, colorectal cancer cell lines express 5–10 times more *ITGA6A* and MYC that their normal counterparts [49] while the integrin α6Aβ4 was found to promote proliferation in colorectal cancer cells [44]. Specific knockdown expression of *ITGA6A* in various CRC cell lines was also accompanied by a reduction in the capacity of these cells to develop tumours [44]. Incidentally, the ability of α6Aβ4 to promote proliferation in cancer cells was found to be mediated by activation of the Wnt/β-catenin pathway [44], a central signalling cascade required for intestinal crypt stem cell homoeostasis that is also exploited by CRC cells [51]. As *MYC* is a Wnt/B-catenin target gene [40], this suggests a feed forward loop for α6Aβ4 and MYC expressions responsible for the overexpression of the two molecules in cancer cells. In this context, it is worth mentioning that experimental overexpression of α6B results in the inhibition of MYC activity [43], an effect potentially mediated by the selective interaction of the MYC inhibitor nucleoshuttling scaffold protein bridging integrator 1 with the α6B cytoplasmic domain [40]. 

## 4. The Integrin α6β4 in CRC

**Figure 3:**
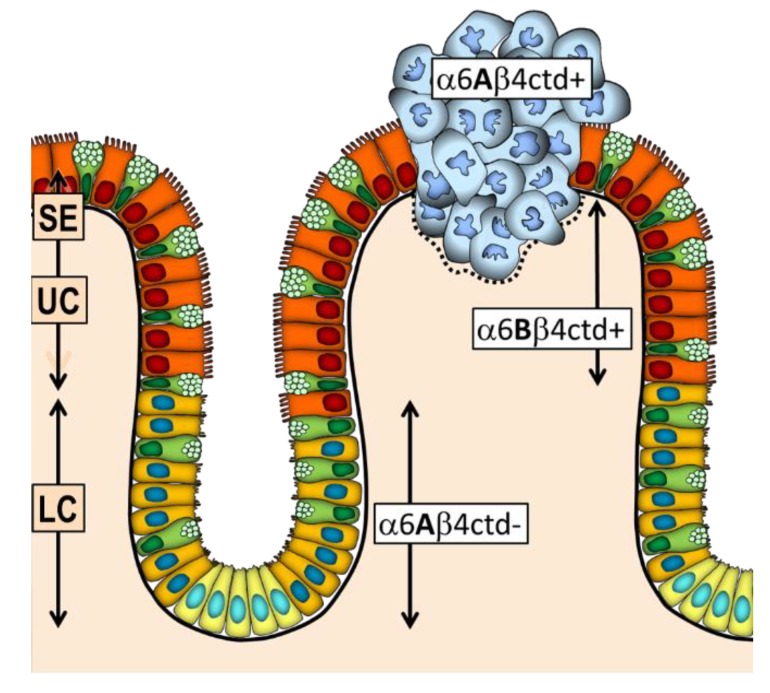
Integrin α6β4 expressed in the human colonic mucosa. In the lower crypt (LC), which contains stem cells (yellow) and proliferative absorptive and goblet cell precursors (gold and light green), α6β4 is expressed under the form of α6Aβ4ctd- while in the upper crypt (UC) and surface epithelium (SE), which contains quiescent absorptive and goblet cells (orange and green), it is under the form of α6Bβ4ctd+. In carcinoma (blue), this segregation is lost and α6β4 is predominantly expressed under the pro-proliferative form α6Aβ4ctd+.

In summary, while there is a clear segregation of α6β4 forms in the normal intestinal mucosa where α6Aβ4cdt- is expressed by the proliferative cells of the lower crypt and α6Bβ4ctd+ is mainly expressed by the quiescent and differentiated cells of the upper crypt and surface epithelium, cancer cells predominantly overexpress α6β4 under the α6Aβ4ctd+ hybrid form (Figure 3), which appears to mediate both cell proliferation and suppression of anoikis [39,52].

## 5. Use of ITGA6 and ITGB4 as Biomarkers for CRC

In light of the functional evidence described above indicating that α6β4 is directly involved in the various steps of CRC progression, there has been growing interest toward assessing its clinical significance as a biomarker for monitoring two critical phases of CRC development: early tumour detection and prognosis. 

### 5.1. Prognostic Factors 

There have been a number of studies that have investigated *ITGA6* and *ITGB4* expression in CRC. Based on the large public datasets that store this information as well as software that allows efficient data mining, it is becoming more and more affordable to analyze these data to compare their expression with pathological features and patient survival. In a recent study, Li et al. [32] analyzed the relation of *ITGB4* level expression in tumours with overall survival using four distinct NCBI GEO datasets as well as the TCGA dataset, selected on the basis of their size (more than 50 patients) and availability of overall survival information. Using the integrated data, high expression of *ITGB4* in CRC was found to be significantly associated with an unfavorable overall survival rate indicating that it is a prognostic factor for colon cancer [32]. *ITGA6* has also been investigated but it was found that its expression did not influence the association of *ITGB4* expression with prognosis in colorectal cancer [32]. However, the *ITGA6A* variant was not specifically considered making the result difficult to interpret. Incidentally, based on the use of genetically modified mouse Itga6 mutant models, De Archangelis et al. concluded that the α6β4 integrin could be classified as a tumour suppressor in the colon based on their observations that depletion of the α6 subunit in intestinal epithelial cells leads to a chronic inflammation that drives the subsequent development of tumors [53], albeit numerous indications that it may drive CRC progression instead, a phenomenon that may be the result of the opposite effect of α6A and B subunit variants on cell functions such as proliferation [54], as summarized above. Consideration of *ITGA6A* and *ITGA6B* separately in future studies should help to clarify these issues.

### 5.2. Screening Factors

Detection of CRC at early stages is a key factor for reducing mortality because this cancer can be treated with high success rates before the occurrence of metastasis [55,56]. Advanced adenomas (AA) are also important to detect since these are considered to be precursors for CRC [57,58]. While colonoscopy remains the gold standard for the detection of colorectal lesions (with sensitivities up to 95% for CRC and 76% for AA), it is an invasive and relatively complex and unpleasant procedure [59]. On the other hand, the fecal immunochemical test (FIT) that detects blood (hemoglobin) in the stools has been used for some time as a non-invasive method but the relatively poor precursor lesion detection rates (66%–80% sensitivity for CRC but less than 28% for AA) limits its utility [60,61] and has triggered exploration of alternate strategies with the potential to improve CRC and AA screening performance. Most of these new strategies are based on non-invasive stool testing for the detection of CRC specific markers and rely on the high rate of intact tumour cells exfoliated into the colon-rectal lumen, a parameter independent of blood release [62,63]. 

**Figure 4:**
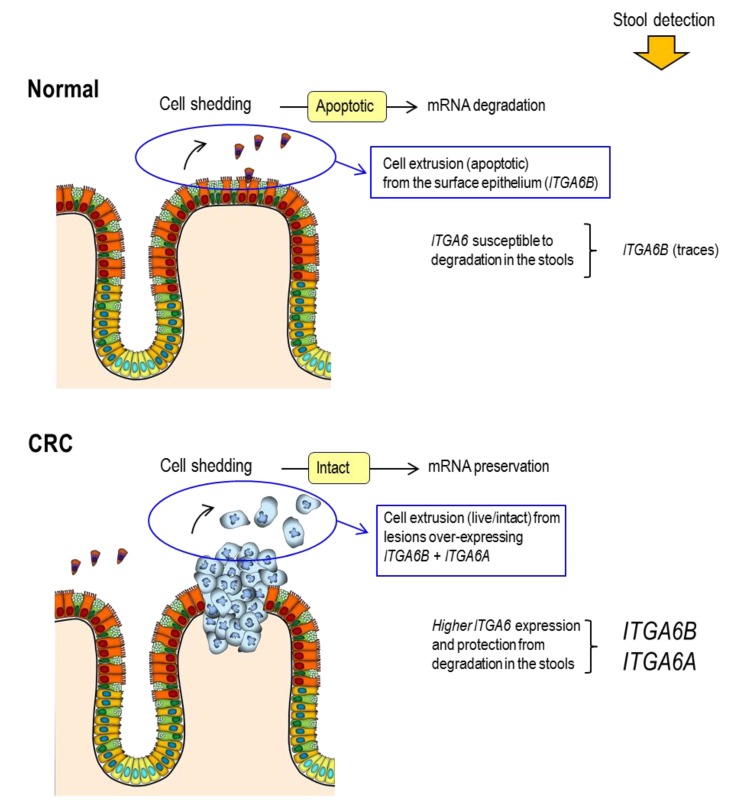
CRC specific markers released by tumor cells exfoliated in the stools: ITGA6. In the normal intestine, cell exfoliation is a regular process by which the oldest epithelial cells become apoptotic and are replaced by new ones produced in the lower crypt. Cell components such as nucleic acids are subject to rapid degradation resulting in a weak accumulation of intact host components in the stools. In contrast, primary tumors protruding into the lumen release a significant number of intact CRC cells that are resistant to apoptosis. Over-expressed cancer cell components such as *ITGA6* are thus likely protected from degradation and can be recovered in detectable levels by standard extraction making them good biomarkers for CRC screening.

While a number of approaches have been proposed over recent years to test released colorectal cancer cell components, mostly nucleic acid, their impact on the improvement of CRC diagnosis has been modest. For instance, the only other FDA approved CRC screening test, Cologuard © which combines the detection of mutations on specific DNA segments with FIT data, improves only slightly lesion detection (92% for CRC and 42% for AA) vs. FIT alone [64]. In a recent study, Barnell et al. [65] used an enrichment protocol for the isolation of RNA from exfoliated colonocytes and high throughput analysis, attaining 45% sensitivity for the detection of AA with a panel of selected targets. Stool mRNA for colorectal lesion screening has been successfully studied over the last decade [66,67]. Considering the up regulation of *ITGA6* in cancer cells, notably under its *ITGA6A* form, our group has also evaluated this target in the stools of patients with colorectal lesions, based on the rationale that exfoliated cancer cells express higher levels of *ITGA6* and are resistant to anchorage-dependent apoptosis, increasing the likelihood of detecting it in the stools (Figure 4).

Using a relatively straightforward protocol for RNA extraction from the stools of patients with AA (24), CRC (91) and controls (60) [68], TaqMan-based quantitative polymerase chain reaction (qPCR) with human specific primers for *ITGA6* and *ITGA6A* confirmed the usefulness and reliability of the approach [69]. Indeed, *ITGA6* levels were found to be increased in stool samples of AA and CRC at all stages while receiver operating characteristic (ROC) curves revealed that *ITGA6* as a single marker can predict 75% of the AA and 81% of the CRC, with a 88% specificity [69]. Comparable results were also obtained using droplet digital PCR [70]. Interestingly, increases in stool *ITGA6A* levels were only found in samples from patients with CRC, consistent with previous observations in primary tumours [43,44]. Combining multiplex qPCR to include additional targets with *ITGA6* in algorithm-based analyses should strengthen this approach toward the development of a new tool for CRC screening.

## 6. Conclusions

Since its discovery as being predominantly expressed in primary CRC, integrin α6β4 has been the subject of various studies that have shown that this laminin receptor can mediate pro-migratory, pro-proliferative and pro-survival properties to cancer cells, three characteristics associated with CRC progression. Incidentally, new studies have shown that *ITGA6* and *ITGB4* have clinical relevance as biomarkers for a stool-based non-invasive CRC screening test and as indicators of overall survival, respectively. Further studies should contribute to implementing these data in the clinical care area, ITGA6 and ITGB4 representing potential therapeutic targets as well as biomarkers for assessing therapeutic therapies.
